# Metabolic dysfunction over a life course key to healthy ageing inequality

**DOI:** 10.1007/s40520-025-03034-3

**Published:** 2025-06-23

**Authors:** Katie Littlewood, Jasleen Gegic, Mike Hickman, Richard C. J. Henson, Jaime R. Bishop, Tim Kershaw, Patrick Diamond, Greg Slabaugh, Emmanouil Tranos, Aphrodite Vasilaki, Daniel Tennant, Emilie Courtin, Li F Chan, Sian M Henson, on behalf of the CELLO Network, Gareth Ackland, Dunja Aksentijevic, William Alazawi, Manuela Angioi, Sara Banks, Michael Barnes, Christopher G. Bell, Katiuscia Bianchi, Joanna Brown, James Buchanan, Livia Carvalho, Jean-Baptiste Cazier, Emma S. Chambers, Claudia Cooper, Laura Cornelsen, Fenn Cullen, Gabor Czibik, Sabrina Diano, Niharika Duggal, Joy Edwards-Hicks, Sarah Finer, Stavros Fotiadis, Lorna Harries, Matina Iliodromiti, Masoud Isanejad, Susan Jarvis, Cath Jenson, Susan Kay, Milan Kiss, Cecilia Lai, Catherine Lester, Kesson Magid, Anne McArdle, Peter McCormick, Alex McKeown, Ira Milosevic, Satomi Miwa, Dylan Morrissey, Rajarshi Mukherjee, Nini Nguyen, Manish Pareek, Fatima Perez de Heredia, Cristina Perez Ternero, Huy Phan, Zudin Puthucheary, Sheena Ramsey, Aivaras Ratkevicius, Yvonne Reinwald, Juan Carlos Rivillas-Garcia, Krisztina Rudolf, Paul Russ, Zafia Salam, Paul Scherer, Johannes Schroth, Carl Sheridan, Egle Solito, Alexandra Stolzing, Samantha Tankard, Stephanie Taylor, Julie Thornton, John Tregoning, Victoria Tsang, Claudia Wilke, Kai Xin Tan, Ruoyan Xu

**Affiliations:** 1 William Harvey Research Institute, Queen Mary University of London, London, EC1M 6BQ UK; 2 Fleet Architects, London, E8 4RG UK; 3 Mile End Policy Institute, Queen Mary University of London, London, E1 4 NS UK; 4 Digital Environment Research Institute, Queen Mary University of London, London, E1 1HH UK; 5 School of Geographical Sciences, University of Bristol, Bristol, BS8 1 TH UK; 6 Institute of Life Course and Medical Sciences, University of Liverpool, Liverpool, L7 8 TX UK; 7 Institute of Metabolism and Systems Research, University of Birmingham, Birmingham, B15 2 TT UK; 8 London School of Economics and Political Science, London, WC2 A 2 AE UK

**Keywords:** Ageing, Metabolism, Health inequalities, Disadvantaged populations

## Abstract

The UK is experiencing a decline in healthy life expectancy, now at 62.4 years for men and 60.9 years for women. Socioeconomic deprivation plays a significant role in health disparities, affecting individuals across the life arc. Girls born in the most deprived areas may live 19 fewer years in good health compared to those in wealthier areas. Health inequalities are particularly severe for ethnic minorities, with Black and Asian individuals reporting poorer health at a younger age. Health inequalities correlate with socioeconomic status. In old age, 2.1 million older adults live in poverty, with Black and Asian communities again disproportionately affected. While ageing increases the risk of morbidities, poor health is not inevitable; however, disadvantaged populations face early-life risk factors, such as low birth weight, linked to future conditions like diabetes and cardiovascular disease. CELLO, an interdisciplinary network, focuses on cellular metabolism throughout life in disadvantaged populations, examining how both genetic and environmental factors shape metabolic dysfunction and contribute to social health inequalities. This review stems from discussions within the network, aiming to advance understanding of healthy ageing across the life course.

**Supplementary Information:**

The online version contains supplementary material available at 10.1007/s40520-025-03034-3.

## Background – healthy ageing inequality

The number of years we spend in good health in the United Kingdom has declined continually from 63.6 years in men and 64.8 years in women in 2008–2010 to 62.4 years for men and 60.9 years for women in 2021–2023 (based on self-reported health data from the UK censuses) [1, 2]. Importantly, the number of years spent in good health varies significantly across the United Kingdom, in accordance with socioeconomic deprivation across the life course. For example, a girl born today in one of the most deprived areas is expected to live 19 fewer years in good health compared to a girl born in one of the least deprived areas [3]. Health inequality is even starker between different ethnic groups, where Asian and Black people in Britain report poor health at a younger age compared to White people. Socioeconomic status contributes to this ethnicity related health disparity where Asian and Black people are twice as likely to live in poverty compared with White people throughout life [4].

Although ageing is itself the most significant risk factor for the appearance of morbidities, frailty and poor health in older adults, the ageing process and mechanisms underpinning this process start before birth and continue through the whole life-arc [5, 6]. Unlike ageing, poor health is not an essential condition of life. But as life begins so does the chance of departing from health. A disproportionately high number of low birth weight babies are born in deprived boroughs, and being born small for gestational age is associated with the development of type 2 diabetes (T2D), hypertension and cardiovascular disease (CVD) at middle age, highlighting the importance of early life in healthy ageing [7]. A key biological factor underpinning social inequalities in healthy ageing are changes in cellular metabolism. To address this, we have established CELLO—an interdisciplinary network focused on *CELLular metabolism Over a life-course* in socioeconomically disadvantaged populations. By centering on metabolism, the network explores how metabolic dysfunction is shaped by both intrinsic (genetic and biochemical pathways) and extrinsic (social and environmental factors) mechanisms, starting from an early age. The primary aim of the CELLO network is to bring together scientists and stakeholders from different disciplines and viewpoints to advance understanding of interdisciplinary methodologies, integrating data analytics to examine the ageing process across the life course in disadvantaged populations, from the cellular level to broader societal impacts.

## Importance of cellular and whole-body metabolism through the life-course

Cellular metabolism operates at the micro level, but its effects scale up to influence population health. Metabolic processes underpin individual health outcomes, shaping susceptibility to chronic diseases like diabetes and cardiovascular conditions [8]. At the population level, metabolic health is influenced by social determinants such as diet, physical activity, socioeconomic status, and environmental exposures [9]. Disparities in these factors contribute to variations in life expectancy and disease burden across different groups. By understanding metabolism in this broader context, we can better inform public health strategies aimed at reducing health inequalities and improving long-term population health outcomes.

**Fig. 1:**
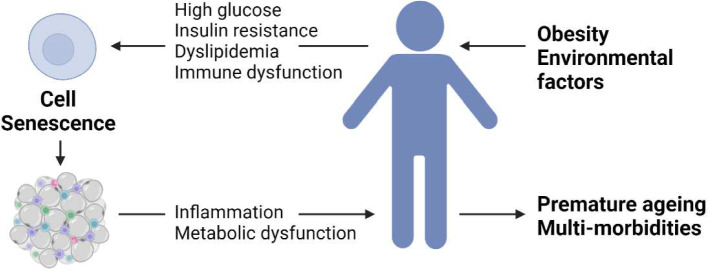
Association between metabolic disease and cellular senescence. The influence of biological, environmental and biopsychosocial factors contributed to the increased senescent cell burden in tissues. Obesity contributes to a diabetic-like state together with immune perturbations, which in turn fuels the accumulation of senescent cells. Senescent cells drive inflammation and metabolic dysfunction, ultimately leading to premature ageing and the appearance of co-morbidities

From a physiological point of view, cellular metabolism comprises the biochemical processes that generate or use energy, taking place within each cell of a living organism. Cellular metabolism encompasses the pathways that are involved in the use of nutrients such as glucose, carbohydrates, fatty acids, and amino acids that are essential for energy homeostasis within the cell [10]. What we eat is incredibly important, with high BMI and obesity directly accelerating the ageing process through undesirable cellular metabolic function. Metabolic dysfunction is often seen in ageing cells, regardless of the type of cell [11]. Mitochondrial dysfunction can drive senescence, a cellular response that limits proliferation, by disrupting the cytosolic NAD^+^/NADH ratios and increasing the generation of reactive oxygen species (ROS) production. Oxygen levels also impact the development of senescent cells, with increased oxygen levels favouring senescence. Hyperglycaemia can also drive senescence in a self-reinforcing mechanism where senescent cells also act as drivers of metabolic disease. Senescent cells accelerate atherosclerotic plaque formation, promote steatosis and hyperinsulinemia. Additionally, senescent cells promote sarcopenia that influences basal metabolism, activity levels, and frailty [11]. When taken together, metabolic dysfunction can be a better predictor of unhealthy ageing than chronological age [12, 13] (Fig. 1).

Cellular metabolism typically focuses on individual metabolic pathways and their regulation in terms of kinetic control by individual reactions within the pathway [14]. While studying cellular metabolism is useful for *in vitro* experiments with controlled conditions, it falls short for understanding *in vivo* metabolism, where cells exist within the dynamic environment of the surrounding tissue [14]. Additionally, the many different metabolic pathways in each individual cell must function coherently. For individual cells to survive, each pathway must be synced and balanced with every other pathway to form an integrated whole. Therefore, a comprehensive understanding of human metabolism requires an integrative analysis of cellular metabolism that also takes into account the context of the entire body [15].

**Fig. 2:**
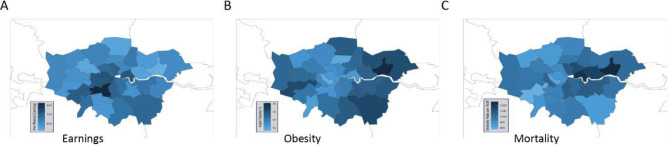
Significant health disparities across London's boroughs, particularly highlighting the stark differences in average weekly earnings, obesity rates, and life expectancy. The data show that poorer boroughs, characterised by lower average weekly earnings (lightest blue), tend to have higher mortality rates and a greater burden of obesity (darkest blue) [78]

Whole-body metabolism and its dysfunction play a key role in many systemic diseases. Metabolic disorders occur when there are changes in extracellular nutrients or deficiencies in key metabolic enzymes, which lead to the accumulation or deficiency of one or more metabolites [16]. Metabolic disorders are associated with external factors, such as an unhealthy lifestyle characterised by low physical activity and excessive calorie intake. Among these, metabolic syndrome (defined as the co-occurrence of impaired fasting glucose, obesity, high blood pressure and dyslipidemia) is the most prevalent being closely associated with the onset of cardiovascular diseases and type 2 diabetes (T2DM). Socioeconomic disadvantage is also linked to metabolic syndrome and research has shown that individuals with limited socioeconomic resources in childhood are more likely to have a higher BMI and an elevated risk of obesity in adults [17] (Fig. 2). This is explained by inequalities in the determinants of weight gain both before and during adulthood, such as physical activity and diet, despite policies designed to reduce them [18]. Furthermore, a notable paradox of the modern food system is that obesity can co-exist with food insecurity. Unhealthy foods are often cheaper per calorie than healthy options, meaning that households unable to afford nutritious diets may also struggle with overall food scarcity [19]. In England, more than half of people over age 45 are living with diet-related health conditions, placing a significant strain on NHS resources [19].

To model the effects of how diet and metabolic syndrome leads to poor health outcomes in socioeconomic disadvantaged populations, preclinical studies are needed. However, diets mimicking human dietary habits are difficult to recapitulate. For example, researchers often use diet-induced obesity animal models, as feeding models reproduce human obesity with greater reliability compared to genetic models [20]. High-fat diets are commonly used to induce obesity in animals with the main lipid source being saturated fatty acids, such as corn oil, peanut oil, soybean oil, sunflower oil, and lard. However, there is no uniform standard for the feed composition used in high-fat diets. Reports have shown that different dietary fats have markedly different impacts on physiological markers and the gut microbiome [21]. The use of lard and palm oil, which contain higher amounts of saturated fatty acid, are relatively unhealthy, negatively affecting the intestinal microbiota and body weight. In contrast, the higher levels of unsaturated fatty acids in fish oil and olive oil are considered relatively healthy, suggesting that various dietary fats contribute differently to weight gain and alterations in the gut microbiome. This is important as the dietary habits of individuals are not homogenous and vary by economic factors, ethnicity, generation, geographic origin, age and religion. The exclusive use of high-fat diets in animal models also misses its interaction with fructose, as a typical Western diet is also high in sugar sweetened drinks containing sucrose and high fructose corn syrup. Higher fructose intake has been observed in people with lower socioeconomic status [22] and the addition of fructose in a high-fat diet leads to the development of obesity, glucose intolerance, and enlarged livers in mice. In contrast, incorporating glucose into a high-fat diet does not produce these effects, even though the calorie intake remains similar suggesting specific negative effects of fructose [23]*.* Understanding how dietary choices interact with socioeconomic position and health outcomes may be the key to minimise premature ageing within low-income areas [22].

## Metabolic dysregulation, accelerated ageing and socioeconomic status

There are many different components that contribute to the development of metabolic disorders. They can be biological, caused by hormonal dysregulation and the generation of adipose tissue [24], or environmental and behavioural, such as having a sedentary lifestyle or the ready availability of fast foods [24]. The connecting factor between these contributing factors is their potential to modify the epigenetic landscape adding to a persistent dysregulated metabolic phenotype. Metabolic intermediaries provide the substrates for epigenetic modifications, including histone modification via methylation, acetylation, and phosphorylation, together with DNA and RNA methylation [25]. However, it’s not just metabolites that can regulate epigenetic modifications, metabolic enzymes can act independently of their metabolic functions via nuclear translocation and the production of chromatin modifying substrates [26]. Therefore, cellular metabolism can also directly communicate environmental changes to the chromatin state.

Metabolic disorders are well documented to accelerate biological ageing [25]. Numerous studies have shown mechanistic causation beyond association [27–31]. For example, shorter leukocyte telomere lengths to be associated with higher triglycerides and fasting glucose, lower high-density lipoprotein cholesterol, and obesity [32, 33], indicating that the severity of the metabolic risk profile is associated with accelerated biological ageing. Therefore, tools that estimate relative epigenetic ageing speed have become valuable as predictors of an individual's health status [34]. The use of epigenetic clocks has also shown a high metabolic score correlates with a faster rate of epigenetic ageing [35–38]. Socioeconomic position is also a major determinant of health outcomes and one prominent idea is that the socioeconomic environment may alter gene expression and subsequent long-term health through changes in the epigenome [39]. Research has indicated that low socioeconomic status is particularly detrimental when experienced during early development and persistently throughout childhood [40].

There is growing interest in the gut microbiota as the link between low socioeconomic status and poor health [41]. Epigenetic mechanisms can also be regulated by the gut microbiota and the cross talk between microbial metabolites, diet and antibiotics contribute to the modulation of metabolic disorders [42]. Protein, fats, digestible and non-digestible carbohydrates, probiotics, and polyphenols all induce changes to the microbiome with secondary effects on host immune and metabolic markers. An acute change in diet, switching to a plant or animal-based diet can alter microbial composition within just 24 hours [43]. Administration of a high-fat, high-sugar diet, leads to a decreased microbial load together with a loss in diversity. The gut microbiome of patients with T2D exhibits microbiota dysbiosis, characterized by reduced bacterial chemotaxis, butyrate synthesis, and the metabolism of cofactors and vitamins [44]. Studies involving microbiota transplantation from obese to lean mice have demonstrated that the obese phenotype is transmissible and associated with microbiota that possess an enhanced ability to extract energy from the host diet [45]. Furthermore, food additives, which are commonly found in processed foods, may impact metabolism by altering the microbiota and inducing changes in the intestinal mucus layer, leading to low-grade inflammation [46]. Therefore, maintaining a healthy gut microbiome is essential for overall human health.

Eating healthily is unaffordable for many and the composition of the microbiota is also influenced by socioeconomic position. In particular, where you live, the amount of greenspace, exposure to pollution, stress, and the type of diet consumed, such as ultra-processed food, governs the variety of microbiota. The level of microbial diversity was lower in low socioeconomic status communities, who showed less resilience and were more prone to pathologies [47] Micronutrients that benefit the microbiota are primarily found in fresh foods, but these nutrients are often lost in the energy-dense, nutrient-poor diets more commonly consumed by those with lower socioeconomic status [48].

## External factors that can influence health and lifespan potential

In addition to studying the physiological homeostasis of the body it is important to understand the extrinsic, biopsychological determinants that enable people to age healthily. Wellbeing is a complex multi-dimensional concept, defined as good mental states, including all of the various evaluations, positive and negative, that people make of their lives, and the affective reactions of people to their experiences [49]. Wellbeing has important bearings on the trajectories of ageing, where high levels of wellbeing are associated with reduced neuro-endocrine, inflammatory, and cardiovascular dysregulation [50].

Social determinants of ageing have a major influence on a person’s health, wellbeing, and quality of life. Where you live, your exposure to racism and crime, educational and employment opportunities, your income, together with access to nutritious foods and physical activity opportunities all have an impact on ageing [51]. These social determinants contribute to health disparities and inequities. Deprived areas have up to 5 times the number of fast-food outlets compared to affluent areas [52, 53]. This lack of access to healthy food raises the risk of CVD, diabetes, and obesity, as well as lowering life expectancy. High levels of inactivity are strongly linked to socioeconomic status, particularly factors such as income, education, and local area deprivation. Even low-cost activities like walking are influenced by socioeconomic status, with disparities widening as the cost of activities increases [54, 55]. Efforts to improve public health are also dictated at the local and sub-regional level and could be undermined by decisions taken in central government. For example UK Government ‘austerity’ measures including cuts to public spending and a reduction in social security payments [56], together with the elimination of existing anti-obesity policies in favour of minimizing the burden on businesses and helping consumers through the cost-of-living crisis [57]. Thus, the government has a part to play in this mechanism too, highlighting the vital association between policy, behaviour, and health outcomes. The long-term impact of policy agenda may be destructive for a future ageing population.

## Lessons from long lived communities: mechanisms leading to improved metabolic health and impact of multigenerational living

Long lived communities can provide some answers to the interplay between intrinsic and extrinsic factors that all contribute to better metabolic health and health span. Global outreach to some of these communities was another aim of the CELLO network, to develop mutual learning on factors that dictate metabolic dysregulation and healthy ageing. Blue Zones, are regions with a high concentration of centenarians and individuals who reach old age without major health complications [58], highlight inequalities in ageing. They demonstrate how lifestyle factors such as diet, social connections, and stress management contribute to significantly longer lifespans. However, these benefits are not equally accessible across all populations, leading to disparities in ageing outcomes across different demographics. Blue Zones include Okinawa in Japan, Ikaria in Greece, Loma Linda in California, the mountain area of Sardinia in Italy, and the peninsula of Nicoya in Costa Rica. Research efforts have sought to investigate the factors associated with this longevity. In particular the eating habits of Blue Zone inhabitants, as these have the advantage of being easily quantifiable and applicable on a larger scale [59]. One emerging theme is that Blue Zone centenarians had reduced access to food or intake of calories. Calorie restriction is thought to manipulate ageing by acting as a mild stressor that promotes homeostatic responses through the downregulation of insulin and insulin-like signalling [60]. Many Blue Zone residents lived in an environment that was non-obesogenic but did not produce malnutrition. The longest-lived people from Okinawa and Southern Italy have a centenarian phenotype similar to adults following a calorie-restricted diet with a low protein to complex carbohydrate ratio (1:10) [60, 61]. Furthermore, the microbiomes of centenarians from Japan and Sardinia were found to have a more diverse microbial content than their younger counterparts [62]. However, a universal food pattern shared between the different Blue Zones has not been found. Rather what has emerged is a diet that is evolving, influenced by multiple historical and societal traditions. This suggests that a diet suitable for a specific population may not suit the lifestyle features of another [59].

The interaction between exercise and diet likely plays a more important role in promoting healthy ageing. Blue Zone populations have high levels of physical activity, incorporating movement into their daily routines [63]. Physical activity benefits multiple organ systems, particularly the cardiovascular system, by improving key risk factors such as BMI, blood pressure, and insulin resistance [64]. Additionally exerkines [65] together with extracellular vesicles [66], released during exercise positively impact cardiovascular, metabolic, immune, and neurological health. Exercise also influences hormonal changes associated with ageing, particularly insulin-like growth factor 1 (IGF-1) [67]. Epidemiological studies have consistently demonstrated that circulating levels of IGF-1 levels decrease with age, whereas centenarians always show higher levels of IGF-1 compared to younger individuals [68]. Finally, physical activity has beneficial effects on several molecular and cellular mechanisms related to ageing, including DNA repair, oxidative stress reduction, inflammation control, and improved mitochondrial function [69]. Therefore, a combined approach of physical activity and proper nutrition may promote longevity and overall well-being.

A common feature of Blue Zone areas is that they typically exhibit a variety of advantageous features, such as a favourable climate, lower levels of air pollution, and ample space for agricultural activities, thereby promoting exercise and physical well-being [70]. Considering the design of urban developments throughout the life-course is crucial, encompassing the physical space of towns, cities, and homes to further support these positive attributes and promote overall health and longevity for people with the poorest life expectancy. Yet, older people are one of the most excluded groups living in urban communities.

Additionally, inhabitants of Blue Zones are characterised by strong familial connections and intergenerational relationships, fostering enhanced social networks that reduce loneliness and social isolation, which are integral to health and well-being [70]. A strong sense of belonging further contributes to lower stress levels and improved psychological well-being [71]. In contrast, while multigenerational living is less common in the UK, recent census data indicates a gradual increase, with multi-generational households rising from 1.8% in 2011 to 2.1% in 2021 [72]. This trend is more prevalent among individuals identifying as"Asian, Asian British, or Asian Welsh". However, unlike in Blue Zones, this form of living is often associated with negative factors in the UK, such as poor air quality, overcrowding, and inadequate housing conditions [73].

## Conclusion – research gaps and innovation for the future

Through discussions within the CELLO network, we identified key research gaps that must be addressed to better inform future studies, spanning from basic to translational research through an interdisciplinary lens. These include: (1) The limitations of diet-induced obesity models, which fail to account for variations in dietary intake influenced by socioeconomic status and ethnicity. (2) The underrepresentation of ethnic and socioeconomic diversity in clinical trials and patient samples, which is particularly critical when studying metabolism and metabolomic readouts, as these factors significantly affect metabolic outcomes. (3) Understanding food choices, food deserts and how differences in food usage are often a downstream factor of educational and economic inequalities, and is essential for addressing broader public health challenges. The CELLO website provides an evolving list of datasets that can help analyse these patterns [74], while a static database is available in the supplementary data. Ultimately major innovation will require global co-operation and linked datasets and bioresource, which was noted to be possible with the COVID pandemic. Additionally, CELLO could help with comparative research on ageing and metabolism, different regions have unique lifestyle, environmental, and genetic factors influencing metabolic health. Applying CELLO in multiple countries allows for cross-country comparisons, helping identify universal versus country-specific drivers of healthy ageing.

The integration of intrinsic and extrinsic metabolic determinants is crucial to promote healthy ageing for all over the life course. Most work in this field has however focused on proximal risk factors. A meta-analysis of 48 international cohorts indicated that social disadvantage was associated with a 2.1-year reduction in life expectancy – a larger decrease than obesity and hypertension combined [75]. Recent findings in separate samples and country settings show that upward social mobility is associated with slower biological ageing, suggesting that interventions that promote social mobility might contribute to addressing inequalities in healthy ageing [76]. Furthermore, it will be necessary to enable the latest medical care for everyone. Especially for the emerging interventions such as senolytics which have been shown to prevent or even cure many age-related conditions in preclinical and clinical trials [77]. Additionally, we need to understand how your postcode is intertwined with health outcomes before we start to comprehend how the cell, self and society interact to paint a complete picture of the state of ageing across the UK.

Without enhancements in healthy life expectancy or productivity within the health service, the UK’s health and care costs will rise as the population continues to age. Interventions that promote healthy living and reduce social isolation throughout life have the potential to significantly improve health in later years. The increasing availability of healthcare data across the UK presents a new frontier for ageing research, where AI and data science can offer novel insights into metabolic ageing. By analysing routine healthcare data, we can gain a clearer picture of how metabolism, health inequalities, and ageing are interconnected, bridging the gap between the cell, self, and society.

## Supplementary Information

Below is the link to the electronic supplementary material.

Supplementary file1 (XLSX 73 KB)

## Data Availability

The repository of data sets listed in supplemetary information can also be found on the CELLO website https://www.ukanet.org.uk/cello/.
